# Human Leukocyte Antigens class II (HLA II) gene profile from an admixed population of patients with type 1 diabetes with severe diabetic retinopathy: a nested case-control study in Brazil

**DOI:** 10.1186/s13098-021-00702-y

**Published:** 2021-08-06

**Authors:** Deborah Conte Santos, Luís Cristóvão Porto, Marcela Haas Pizarro, Laura Gomes Nunes de Melo, Dayse A. Silva, Romulo Vianna Oliveira, Anna Paula Villela, Luiza Harcar Muniz, Camila Soares, Lucianne Righeti Monteiro Tannus, Karla Rezende Guerra Drummond, André Araújo Pinheiro, Felipe Mallmann, Franz Schubert Lopes Leal, Fernando Korn Malerbi, Paulo Henrique Morales, Marília Brito Gomes

**Affiliations:** 1 Department of Internal Medicine, Diabetes Unit, Rio de Janeiro State University (UERJ), Boulevard 28 de Setembro, 77-3º andar, Vila Isabel, Rio de Janeiro, Rio de Janeiro CEP 20551-030 Brazil; 2 Histocompatibility and Cryopreservation Laboratory (HLA), Rio de Janeiro State University (UERJ), Rio de Janeiro, Rio de Janeiro Brazil; 3 Department of Ophthalmology, State University of Rio de Janeiro, Rio de Janeiro, Brazil; 4 DNA Diagnostic Laboratory (LDD), Rio de Janeiro State University (UERJ), Rio de Janeiro, Rio de Janeiro Brazil; 5 Department of Ophthalmology, Federal University of São Paulo, São Paulo, Brazil; 6 Department of Ophthalmology, Regional Hospital of Taguatinga, Brasília, Brazil; 7 Department of Ophthalmology, Federal University of Rio Grande Do Sul, Porto Alegre, Brazil; 8 Department of Ophthalmology, University of Campinas, Campinas, São Paulo Brazil; 9 Department of Endocrinology and Ophthalmology, Federal University of São Paulo, São Paulo, Brazil

**Keywords:** Type 1 diabetes, Retinopathy, HLA, Human leukocyte antigen

## Abstract

**Background:**

Although the well-established role of the HLA genes on the predisposition of type 1 diabetes (T1D), its contribution to the development and progression of diabetic retinopathy is still unclear, especially in admixed populations. We aimed to study the relationship between HLA alleles and severe diabetic retinopathy in a highly admixed population of T1D patients.

**Methods:**

This was a nested case-control study based on a cross-sectional, nationwide survey conducted in Brazil. We included 117 patients with severe diabetic retinopathy and 117 random controls composed of T1D patients without retinopathy, matched for diabetes duration. *HLA*-class II genes (*HLA-DRB1, -DQA1, and -DQB1*) were genotyped using the SSO and NGS methods.

**Results:**

Haplotypes HLA-DRB1*04:05 ~ DQA1*03:01 g ~ DQB1*03:02 (OR 1.75, CI 0.97–3.16, p value 0.058) and HLA-DRB1*13:02 ~ DQA1*01:02 ~ DQB1*06:04 (OR 5.18, CI 1.12–23.09, p value 0.019) were more prevalent on the severe DR group but they did not present statistically difference after Bonferroni correction. The most frequent haplotype on both groups was HLA-DRB1*03:01 ~ DQA1*05:01 g ~ DQB1*02:01 (29.6% on severe DR and 33.33% on the control group).

**Conclusions:**

Our study showed no influence of HLA genes on the development of DR. Further longitudinal data is needed to better understand the role of genetic factors on this multifactorial significant microvascular complication.

## Background

Type 1 diabetes (T1D) is a chronic disease that arises from multiple genetic and environmental factors [1]. Although several different loci influence the expression of T1D, the HLA region on chromosome 6p21 responds to almost 50% of the risk [2].

A prevalence of 30% of microvascular complications is observed in T1D patients from different populations [3]. Diabetic retinopathy (DR) is one of the most frequent diabetes-related chronic complications, with an estimated global prevalence of 35%. DR is considered the leading cause of blindness in the adult population resulting in a significant social and financial burden [4]. The diabetes control and complications trial (DCCT) and epidemiology of diabetes interventions and complications (EDIC) studies [5] showed that adequate glycemic control is essential to avoid or postpone diabetes-related chronic complications, including DR.

Besides glycemic control and diabetes duration, HLA-DRB1*04 and HLA-DRB1*03 have been linked to the development and progression of DR with controversial results [6–12]. The WESDR cohort with T1D Caucasian patients showed a positive association of HLA-DRB1*04 with DR in its first cross-sectional analysis with no adjustment for disease duration [13]. In contrast, in its 14-years-follow-up report, no differences were found [6]. Another study conducted in the Caucasian population showed a protective effect of HLA-DRB1*03 and a risk effect of HLA-DRB1*04 [14].

The controversial results found in previous studies could be even more controversial in a highly admixed population, such as the Brazilian. A previous HLA study in Brazilian type 1 diabetes patients showed similar haplotypes frequencies for HLA-DRB1*03 and HLA-DRB1*04 as described in the Caucasian population. However, it also demonstrated the association of the HLA-DRB1*09 allele with T1D, mainly expressed in the African-American population [15]. The heterogeneous Brazilian population is composed of a highly admixed combination of three principal ancestral roots: Native Amerindians (NAM), Europeans (EUR), and Africans (AFR). The process of population admixture started with European colonization (particularly by the Portuguese) in the coastal region, eventually spreading to the country’s interior, which was populated by Native Amerindians. Afterward, with the heavy slavery traffic (particularly from Africa), even more migration and admixture have occurred, explaining the substantial genetic diversity in Brazil.

We aimed to study the relationship between HLA alleles and severe diabetic retinopathy in a highly admixed population of type 1 diabetes patients.

## Methods and subjects

### Study design and population

The present study was a nested case-control study based on a cross-sectional, nationwide survey conducted in Brazil’s five geographic regions between August 2011 and August 2014. The original study included 1,760 patients with T1D, and the methods have been described previously [16]. Briefly, all patients received health care from the Brazilian National Health Care System (SUS). The diagnosis of type 1 diabetes was made based on typical clinical presentation (hyperglycemia, weight loss, polyuria, polydipsia, polyphagia, and the need for continuous insulin use since the diagnosis). Inclusion criteria were: at least 13 years of age and minimum follow-up at each center of at least 6 months.

For the present study, we included 117 cases, defined as patients with severe diabetic retinopathy (severe nonproliferative or proliferative diabetic retinopathy) and 117 random controls defined as patients from the same cohort without retinopathy, matched for diabetes duration by a range of 5 years. The Ethics Committee of the University Hospital of Pedro Ernesto approved the study. Written informed consent was obtained from all participants or their representatives.

Participants responded to a standardized questionnaire during a clinical visit to evaluate clinical and demographic data such as current age, age at diagnosis, gender, duration of diabetes, years of formal education and socio-economic status, and self-reported color/race. They were also submitted to clinical evaluation to assess the presence of complications and the measurement of clinical data. Hypertension was considered if self-reported or in the presence of a health practitioner’s previous diagnosis on at least two separate occasions. Glomerular Filtration Rate (GFR) was estimated by the CKD-EPI equation [17] in adults and by the Schwartz formula [18] in adolescents and was expressed in milliliters per minute per 1.73 m^2^ (ml/min). Blood sampling was collected to determine HbA1c levels (high-performance liquid chromatography-HPLC, Bio-Rad Laboratories, Hercules, California, USA) and genetic analysis.

### Evaluation of diabetic retinopathy

Mydriatic binocular indirect ophthalmoscopy with an Eyetec Ophthalmoscope (Eyetec, São Carlos-SP, Brazil) and a 20-diopter lens (Volk Optical, Mentor, OH, USA) was performed by an experienced retinal specialist in each center. Patients had both eyes examined. Mydriasis was obtained with 1% tropicamide drops. All ophthalmologists who performed the fundoscopy were trained by the same retinal specialist and followed the same protocol. For patient classification, we considered the eye with the most severe classification of DR (absent, mild nonproliferative, moderate nonproliferative, severe nonproliferative, and proliferative DR) according to the American Academy of Ophthalmology guidelines [19].

### DNA extraction and HLA genotyping

Genomic DNA was extracted from peripheral blood with the commercial kit SP QIA symphony by automation with QIA symphony equipment, following the manufacturer’s instructions (Qiagen, USA).

HLA-class II genes (*HLA-DRB1*, *HLA-DQA1*, and *HLA-DQB1*) were genotyped either by Next Generation Sequencing (NGS) or Medium to high-resolution PCR-RSSO (LabType SSO, One lambda Inc. West Hills, USA) method. Detailed information on genotyping of our studied population was described previously [20]. Briefly, in this present analysis, 79 (34%) individuals were genotyped using PCR- RSSO (LabType SSO2B1 High resolution, One Lambda Inc., West Hills, USA) and 155 (66%) patients by next-generation sequencing (NGSgo.v2, GenDx, Utrecht, the Netherlands). We used the Common and Well Documented version 2.0 (CWD2) to assign HLA-DRB1 and HLA-DQB1 genes ambiguities. Three-locus haplotype frequencies (*HLA-DRB1* ~ *HLA-DQA1* ~ *HLA-DQB1*) were estimated using the expectation-maximization (EM) algorithm [21, 22]. Deviations from Hardy–Weinberg equilibrium (HWE) were assessed at the allele-family level (first nomenclature field) using a modified version of the Guo and Thompson algorithm [23] as implemented in the software Arlequin v.3.5 [24].

Ambiguous HLA class II alleles within G group (i.e., groups of alleles that have identical nucleotide sequences across the exons encoding the peptide binding domains) were designated by a lower case ‘g’ (HLA-DRB1*12:01 g  =  12:01/12:10; HLA-DQA1*01:01 g  =  01:01/01:04/01:05; HLA-DQA1*03:01 g  =  03:01/03:02/03:03; HLA-DQA1*05:01 g  =  05:05/05:09; HLA-DQB1*03:01 g  =  *0.03:01/03:09/03:19*).

### Statistical analysis

Categorical variables were presented as frequency (percentage). All normally distributed values were given as the mean  ±  standard deviation (SD), and all other values were given as the median (IQR). We used Chi-squared and Fisher’s tests to compare categorical data; the Student t test and analysis of variance (ANOVA) were used for comparisons between groups with numeric variables when indicated. Samples were divided into two groups (patients with T1D and severe DR and patients with T1D without DR) for population comparison testing. Arlequin software was used to calculate FST genetic distance, and the exact test for population differentiation test results was performed via allele frequencies extrapolations [24]. Bonferroni correction was applied for multiple tests. We used the Statistical Program for Social Sciences version 17.0 (SPSS, Inc., Chicago, Illinois). A two-sided p value of less than 0.05 was considered significant. Haplotype frequencies in cases and controls were compared using a Pearson X^**2**^ test. Odds ratios (ORs) and 95% CIs were computed.

## Results

### Population characteristics

Table 1 shows the study population characteristics. Patients with severe DR were older, with lower GFR, higher levels of HbA1c, and were more prone to hypertension than patients without DR.

**Table 1:** Characteristics of the study participants

Variable	Patients with severe DR, N = 117	Patients without DR, N = 117	*p* value
Sociodemographic			
Gender, N (%)			**0.011**
Male	57 (48.72)	38 (32.48)	
Female	60 (51.28)	79 (67.52)	
Age, y mean (SD)	38.8 (11.68)	34.56 (12.32)	**0.007**
Economic status, N (%)			0.237
High	4 (3.42)	7 (5.98)	
Medium	52 (44.44)	49 (41.88)	
Low	52 (44.44)	58 (49.57)	
Very low	9 (7.69)	3 (2.56)	
Years of schooling, y mean (SD)	11.98 (3.99)	12.50 (3.68)	0.300
Diabetes-related variables			
HbA1c (%), mean (SD)	8.89 (1.87)	8.40 (1.74)	**0.043**
HbA1c mmol/mol, mean (SD)	73.64 (20.41)	68.38 (19.01)	**0.043**
Duration of diabetes, y mean (SD)	23.42 (9.28)	22.18 (8.32)	0.283
GFR	66.62 (24.35)	83.04 (25.60)	**< 0.001**
Arterial hypertension, y, N (%)	50 (42.68)	21 (17.95)	**< 0.001**
Self-reported ethnicity, N (%)			0.320
Caucasian	62 (52.99)	65 (55.55)	
Black	15 (12.82)	9 (7.69)	
Mullatos	36 (30.77)	41 (35.04)	
Native Americans	4 (3.42)	1 (0.85)	
Asian	0	1 (0.85)	

Bold represents statistical significance (*p* < 0.05)

Data are present as number (percentage), mean  ±  SD (standard deviation), or median (IQR or range)

*DR* diabetic retinopathy; *y *years

### Overview of the risk and protective alleles and/or haplotypes of the HLA system in patients with severe DR and controls

Allele frequencies and distribution between groups are described in Table 2. The HLA-DRB1*13:02 allele was more frequent in patients with severe DR (6.41 vs. 1.7, p  =  0.01, OR  =  3.94). HLA-DQB1*03:01 was also more frequent in patients with severe DR (9.40 vs. 4.70, OR  =  2.10, p  =  0.047). None of the HLA-DRB1*, HLA-DQA1*, and HLA-DQB1* genes showed statistically significant differences between groups after Bonferroni correction for multiple tests.

**Table 2:** HLA-DRB1*, -DQB1*, and -DQA1* alleles distribution in patients with severe DR and patients without DR

	Patients with severe DR N = 117		Patients without DR N = 117		OR	(CI)	p
	N	(%)	N	(%)			
HLA-DRB1*							
01:01	6	(2.56)	8	(3.42)	0.74	(0.25–2.18)	0.59
01:02	5	(2.14)	6	(2.56)	0.83	(0.25–2.76)	0.76
03:01	68	(29.06)	80	(34.19)	0.79	(0.53–1.16)	0.23
04:01	8	(3.42)	15	(6.41)	0.52	(0.21–1.24)	0.13
04:02	13	(5.56)	14	(5.98)	0.92	(0.42–2.01)	0.84
04:04	8	(3.42)	10	(4.27)	0.79	(0.31–2.05)	0.63
04:05	35	(14.96)	24	(10.26)	1.54	(0.88–2.68)	0.13
07:01	18	(7.69)	23	(9.83)	0.76	(0.40–1.46)	0.41
09:01	4	(1.71)	10	(4.27)	0.39	(0.12–1.26)	0.10
** 13:02**	15	(6.41)	4	(1.71)	3.94	(1.29–12.05)	**0.01**
Others	54	(23.08)	40	(17.09)	1.45	(0.92–2.30)	0.11
HLA-DQA1*							
01:01 g	17	(7.26)	21	(8.97)	0.79	(0.41–1.55)	0.49
01:02	24	(10.26)	15	(6.41)	1.67	(0.85–3.27)	0.13
01:03	5	(2.14)	3	(1.28)	1.68	(0.40–7.12)	0.48
02:01	17	(7.26)	22	(9.40)	0.75	(0.39–1.46)	0.40
03:01 g	74	(31.62)	77	(32.91)	0.94	(0.64–1.39)	0.77
04:01	12	(5.13)	6	(2.56)	2.05	(0.76–5.57)	0.14
04:02	1	(0.43)	0	(0.00)			NA
05:01 g	82	(35.04)	90	(38.46)	0.86	(0.59–1.26)	0.44
05:10	2	(0.85)	0	(0.00)			NA
HLA-DQB1*							
02:01	71	(30.34)	81	(34.62)	0.82	(0.56–1.21)	0.32
02:02	23	(9.83)	35	(14.96)	0.62	(0.35–1.09)	0.09
** 03:01 g**	22	(9.40)	11	(4.70)	2.10	(1.0–4.44)	**0.047**
03:02	60	(25.64)	58	(24.79)	1.05	(0.69–1.59)	0.83
04:02	11	(4.70)	5	(2.14)	2.26	(0.77–6.61)	0.13
05:01	17	(7.26)	20	(8.55)	0.84	(0.43–1.64)	0.61
06:04	10	(4.27)	4	(1.71)	2.57	(0.79–8.30)	0.1
Others	20	(8.55)	20	(8.55)	1.00	(0.52–1.91)	1

Bold represents statistical significance (*p* < 0.05)

Rare alleles were included in others

*DR* diabetic retinopathy; *N *number of individuals; *OR *odds ratio; *CI *confidence interval; *NA *non applicable

Table 3 shows the distribution of the *HLA-DRB1* ~ *DQA1* ~ *DQB1* haplotypes in patients with severe DR and patients without DR. The most frequent haplotype on both groups was HLA-DRB1*03:01 ~ DQA1*05:01 g ~ DQB1*02:01 (29.6% on severe DR and 33.33% on patients without DR group). Haplotypes HLA-DRB1*04:05 ~ DQA1*03:01 g ~ DQB1*03:02 (OR 1.75, CI 0.97–3.16, p value 0.058) and HLA-DRB1*13:02 ~ DQA1*01:02 ~ DQB1*06:04 (OR 5.18, CI 1.12–23.09, p value 0.019) were more prevalent on the severe DR group but they did not present statistically difference after Bonferroni correction.

**Table 3:** Distribution of the *HLA-DRB1* ~ *DQA1* ~ *DQB1* haplotypes in patients with severe DR and patients without DR

Haplotype	Patients with severe DR N = 117		Patients without DR N = 117		OR	(CI)	p
*HLA-DRB1* ~ *DQA1* ~ *DQB1*	N	%	N	%			
*03:01* ~ *05:01 g* ~ *02:01*	68	(29.06)	78	(33.33)	0.82	(0.55–1.21)	0.32
*04:05* ~ *03:01 g* ~ *03:02*	33	(14.10)	20	(8.55)	1.75	(0.97–3.16)	0.06
*07:01* ~ *02:01* ~ *02:02*	16	(6.84)	21	(8.97)	0.74	(0.38–1.46)	0.39
*04:02* ~ *03:01 g* ~ *03:02*	13	(5.56)	13	(5.56)	1.00	(0.45–2.21)	1
***04:01* ~ *03:01 g* ~ *03:02***	5	(2.14)	13	(5.56)	0.37	(0.13–1.06)	**0.05**
*04:04* ~ *03:01 g* ~ *03:02*	7	(2.99)	9	(3.85)	0.77	(0.28–2.11)	0.61
*09:01* ~ *03:01 g* ~ *02:02*	4	(1.71)	8	(3.42)	0.49	(0.15–1.65)	0.24
***13:02* ~ *01:02* ~ *06:04***	10	(4.27)	2	(0.85)	5.18	(1.12–23.90)	**0.02**
*01:01* ~ *01:01 g* ~ *05:01*	5	(2.14)	6	(2.56)	0.83	(0.25–2.76)	0.76
Others	73	(31.20)	64	(27.35)	1.2	(0.81–1.80)	0.36

Bold represents statistical significance (*p* < 0.05)

Rare alleles were included in others

*DR*  diabetic retinopathy; *N *number of individuals; *OR *odds ratio; *CI *confidence interval

*HLA-DRB1/HLA-DRB1* genotypes are demonstrated in Table 4. The most frequent genotype on both groups was HLA-DRB1*03/HLA-DRB1*04 (26.5% on each group). The HLA-DRB1*13/X was more prevalent in the severe DR group (OR 5.37, CI 1.15–25.09, p value 0.019). Although HLA-DRB1*09/X and HLA-DRB1*03/X were more frequent in the group of patients without DR, this difference was not statistically significant.

**Table 4:** HLA-DRB1*/HLA-DRB1* genotypes distribution in patients with severe DR and patients without DR

HLA-DRB1*/HLA-DRB1*	Patients with severe DR N = 117		Patients without DR N = 117		OR	(CI)	p
	N	(%)	N	(%)			
HLA-DRB1*03/HLA-DRB1*03	13	(11.11)	15	(12.82)	0.85	(0.38–1.87)	0.69
HLA-DRB1*03/HLA-DRB1*04	31	(26.50)	31	(26.50)	1.00	(0.56–1.79)	1.00
HLA-DRB1*03/X	10	(8.55)	16	(13.68)	0.59	(0.27–1.36)	0.21
HLA-DRB1*04/HLA-DRB1*04	6	(5.13)	5	(4.27)	1.21	(0.36–4.09)	0.76
HLA-DRB1*04/X	20	(17.09)	20	(17.09)	1.00	(0.51–1.97)	1.00
HLA-DRB1*09/HLA-DRB1*03	3	(2.56)	4	(3.42)	0.74	(0.16–3.40)	0.70
HLA-DRB1*09/HLA-DRB1*04	0	(0.00)	1	(0.85)			NA
HLA-DRB1*09/HLA-DRB1*09	0	(0.00)	0	(0.00)			NA
HLA-DRB1*09/X	1	(0.85)	5	(4.27)	0.19	(0.02–1.68)	0.10
HLA-DRB1*13/HLA-DRB1*13	0	(0.00)	0	(0.00)			NA
HLA-DRB1*13/HLA-DRB1*03	3	(2.56)	1	(0.85)	3.05	(0.31–29.78)	0.31
HLA-DRB1*13/HLA-DRB1*04	7	(5.98)	5	(4.27)	1.42	(0.44–4.63)	0.55
*HLA-*DRB1*13/X	10	(8.55)	2	(1.71)	5.37	(1.15–25.09)	**0.02**
DRX/DRX	13	(11.11)	12	(10.26)	1.09	(0.48–2.51)	0.83

Bold represents statistical significance (*p* < 0.05) before Bonferroni correction

p value required for significance after Bonferroni correction 0.004

*DR *diabetic retinopathy; *N *number of individuals; *OR *odds ratio; *CI *confidence interval; *NA *non applicable; *DRX *any haplotype other than HLA-DRB1*03, HLA-DRB1*04, HLA-DRB1*09 or HLA-DRB1*13

## Discussion

Our study, conducted in a highly admixed population with T1D, showed no influence of HLA genes on the development of DR. All the possible risk associations such as those with the HLA-DRB1*13 allele and with the haplotype HLA-DRB1*13:02 ~ DQA1*01:02 ~ DQB1*06:04 as well the protection association such as that with the haplotype HLA-DRB1*04:01 ~ DQA1*03:01 g ~ DQB1*03:02 were no longer significant after Bonferroni correction. Independent of the retinopathy status, HLA-DRB1*03 and HLA-DRB1*04 were the most prevalent alleles.

Although HLA's role in the predisposition to T1D has been studied for decades [25–28], whether the HLA region influences the development of microvascular complications, especially retinopathy, is a matter of some controversy [6–9, 12–14, 29]. Several studies from different populations, including Brazilians, showed that HLA-DRB1*03 and HLA-DRB1*04 are the most frequent alleles in patients with type 1 diabetes [15, 28]. A recently published paper from our group using a large number of patients from Brazil also found those alleles as the most prevalent in the T1D group [20]. The present study and other studies in diabetic retinopathy demonstrated the HLA-DRB1*03 and HLA-DRB1*04 alleles as the most prevalent. Although some studies found the HLA-DRB1*04 allele associated with the risk of developing DR and the HLA-DRB1*03 allele to be related to protection [6], others found no association when comparing patients with severe DR and patients without severe DR [11–13]. Our study found no association between groups after Bonferroni corrections. It is important to note that most of the previous studies did not adjust for critical classic factors associated with DR, such as duration of diabetes, and were conducted in Caucasian populations.

Genetic predisposition to DR might be influenced by the differences in ancestry profiles of the studied populations. A recent study from our group with a similar group of patients and controls showed a difference between groups' ancestry profiles [30]. African genomic ancestry was associated with DR even after corrections. This could indicate that genetic predisposition might be related to other non-HLA genes.

In addition, the lack of association of the HLA genes and the development of DR might rely on the basis of DR’s pathogenesis. The pathogenesis of DR is multifactorial and still not yet completely understood. One well-established mechanism is the endothelial lesion mediated by hyperglycemia or hypoxia. High glucose levels induce oxidative stress mediated by the reduced effect of NO and activation of macrophages and the productions of inflammatory cytokines [31]. The TNF alfa has its gene located in the same region of HLA and has been implicated in the pathogenesis of DR [32], but genetic studies of its relation with DR predisposition are yet to be proved [33, 34]. Different genes have been studied in search of DR's genetic predisposition, but the only ones that showed relevant results were the polymorphisms of aldose reductase (AKR1B1) and VEGF genes [34]. Recent studies have demonstrated neurodegeneration as an early factor in the pathogenesis of DR, and some other studies have concentrated on the role of epigenetics, especially in mitochondrial DNA [35]. Despite several treatment options for severe DR, some patients do not achieve a satisfactory response to treatment. Therefore, further research is needed to better elucidate novel mechanisms related to this multifactorial disease.

Duration of diabetes is a critical factor in the development of DR, as demonstrated in previous data from several studies, including a survey from our group [36]. It is hypothesized that HLA-related influence on DR might be significant only in patients that develop DR in the early course of T1D [12]. In our study, as we matched patients and controls by the duration of diabetes in a 5-years range, therefore, excluding this potential confounder from our analysis, this time-influenced association might be diminished. However, this hypothesis is yet to be proved by further extensive longitudinal data.

Our study has strengths and limitations. One particular strength is the population-based ascertainment of diabetes cases in a highly admixed population. Also, we adjusted for the duration of diabetes, one of the most important risk factors for DR, at selection, matching controls by a range of five years. Our study was based only on patients assisted by the public health system in urban areas. Although this could have led to some selection bias, most T1D patients are treated on the public health system or both public and private health care in Brazil. Another limitation is that autoantibodies and C-peptide levels were not measured. This misdiagnosis bias might be mitigated by the fact that 96.5% of the patients included were diagnosed before 30 years of age. Also, the diagnosis of DR was assessed by binocular indirect ophthalmoscopy for all participants. Despite the inherent limitations of this method, a previous study from our group showed a substantial agreement between binocular indirect ophthalmoscopy and digital retinography [37].

In conclusion, our study, performed in a highly admixed population comparing patients with severe DR to T1D without DR matched for diabetes duration, found no association between *HLA-Class II* genes and severe DR. Further longitudinal data is needed to better understand the role of genetic factors on this multifactorial significant microvascular complication.

## Data Availability

Data will be made available upon request.

## Abbreviations

TID: Type 1 diabetes
HLA: Human leukocyte antigen
DCCT: Diabetes control and complication trial
EDIC: Epidemiology of diabetes interventions and complications
DR: Diabetic retinopathy
NAM: Native Amerindian
EUR: European
AFR: African
GFR: Glomerular filtration rate
NGS: Next generation sequencing
SD: Standart deviation
IQR: Inter quartil range
NO: Nitric oxide
TNF: Tumor necrosis factor
VEGF: Vascular endothelial growth factor
